# Intercostal nerve cryoablation therapy for the repair of pectus excavatum: a systematic review

**DOI:** 10.3389/fsurg.2023.1235120

**Published:** 2023-08-24

**Authors:** R. Scott Eldredge, Lisa McMahon

**Affiliations:** Department of Surgery, Division of Pediatric Surgery, Phoenix Children’s Hospital, Phoenix, AZ, United States

**Keywords:** pectus excavatum, minimally invasive repair of pectus excavatum, cryoablation, Nuss, cryoanalgesia

## Abstract

**Introduction:**

The minimally invasive repair of pectus excavatum (PE) is a painful procedure that can result in long-term hospitalization and opioid use. To mitigate the length of stay and opioid consumption, many different analgesia strategies have been implemented. The aim of this study is to review the use and patient outcomes of intercostal nerve cryoablation (INC) during PE repair reported in the literature.

**Methods:**

An unfunded literature search using PubMed identifying articles discussing INC during PE repair from 1946 to 1 July 2023 was performed. Articles were included if they discussed patient outcomes with INC use during PE repair. Articles were excluded if they were reviews/meta-analyses, editorials, or not available in English. Each article was reviewed for bias by analyzing the study methods, data analysis, patient selection, and patient follow-up. Articles comparing outcomes of INC were considered significant if *p*-value was <0.05.

**Results:**

A total of 34 articles were included in this review that described INC use during pectus repair. Most supported a decreased hospital length of stay and opioid use with INC. Overall, INC was associated with fewer short-term and long-term complications. However, the researchers reported varied results of total hospital costs with the use of INC.

**Conclusion:**

The review was limited by a paucity of prospective studies and low number of patients who received INC. Despite this, the present data support INC as a safe and effective analgesic strategy during the repair of PE.

## 1. Introduction

Pectus excavatum (PE) is the most common chest wall deformity characterized as an inward depression of the sternum, affecting one in every 250 adults with a female predominance of 5:3 (1–3). The sternal depression is hypothesized to be secondary to inward overgrowth of the costal cartilage, which is commonly exacerbated during puberty (4, 5). PE may have a myriad of adverse effects, ranging from impaired cardiopulmonary performance during rest and exercise to poor psychosocial outcomes (2).

The current gold standard for the repair of PE is the minimally invasive repair of PE (MIRPE), or the Nuss procedure, which has smaller incisions and decreased operative time and blood loss when compared with an open chest wall reconstruction, or the Ravitch procedure. MIRPE is a very safe procedure when performed in combination with a sternal elevation and intrathoracic visualization but is associated with more pain than the Ravitch procedure (6–8). In an attempt to mitigate patients pain following MIRPE, many analgesic strategies have been proposed including the use of thoracic epidurals (TEs), intravenous patient-controlled analgesia (PCA), indwelling chest wall catheter infusion or elastomeric pain pumps (EPPs), and local or regional nerve blocks (9–13).

The use of intercostal nerve cryoablation (INC) as an analgesic adjunct during the MIRPE was first reported in 2016 by Keller et al. (14) when they found that the use of INC was associated with a decreased length of stay (LOS) and inpatient opioid consumption when compared with TE. INC is thought to have temporary neurosensory effects and takes advantage of the ability of the peripheral nerves to regenerate following injury (15–17). Since the introduction of INC during the MIPRE, many surgeons have adopted this technique. The aim of our study is to review the reported patient outcomes of those who had undergone INC during PE repair in the current literature.

## 2. Methods

A literature search was performed using “Cryoablation” or “Cryotherapy” and “Pectus Excavatum” using PubMed from 1946 to 1 July 2023. All titles and abstracts were reviewed for content and subject relevance. Articles were excluded from the review if they did not pertain to patient outcomes of PE repair with the use of INC, if the article was not available in English, if the article was a review or meta-analysis, or if the article was an opinion piece. In addition, the citations were reviewed for all included articles. If a cited article was identified that pertained to INC during PE repair, it was then included in the review.

Two reviewers screened all the articles for the inclusion and exclusion criteria; upon selection, each article was reviewed, and data were abstracted pertaining to the study methods, patient demographics, INC technique, operative duration, INC comparison group, patient LOS, inpatient and outpatient oral morphine equivalence (OME), patient-reported pain scores, hospital charges, and surgical complications. The details pertaining to INC were recorded including number of nerves and intercostal spaces cryoablated and the duration and temperature of nerve cryoablation. The operative duration was recorded as both surgical time and operating room time if reported. The patient-reported pain scores were recorded on a Likert scale from 1 to 10. All complications reported by the authors were abstracted. The data points were excluded in this review if they were not reported by the authors or if any data points were unclear.

An in-depth assessment of articles discussing the primary outcome of LOS and secondary outcomes among patients who had undergone INC vs. a control analgesic strategy was conducted. Both prospective and retrospective studies were included in this review. Comparisons of outcomes were abstracted between study groups; outcomes between groups were considered statistically significant if a *p*-value of <0.05 was reported. All comparisons of LOS, opioid usage, and pain scores were compiled in a table regardless of statistical significance. The patient demographics were reviewed between those who received INC and those who received a different analgesic strategy to ensure patient similarities between groups. To reduce bias, the authors of this manuscript independently reviewed each study that was identified using PubMed for the inclusion criteria.

## 3. Results

A total of 44 articles were identified via the defined literature search (33) and article citation review (5); of these articles, 34 were included in our review. Of the 10 excluded articles, four did not pertain to INC outcomes following the MIRPE (18–21), three were opinion editorials (22–24), two were review articles (25, 26), and two were not in the English language (27). Of the articles included, the majority were single-center retrospective reviews (29/34), with one randomized control trial and four prospective reviews. A total of 47% of the articles included both pediatric and adult patients in their analysis; however, the majority of the patients were pediatric, ≤18 years old, with an average age of less than 21 years in all articles. Most articles contained fewer than 60 patients who had undergone INC, and the largest study contained 350 patients. A majority (24/34) compared patient-related outcomes between INC and a control group. The control groups included multimodal pain regimen, thoracic epidural PCA, paravertebral nerve block with and without continuous infusion, elastomeric pain pump, or unspecified analgesia strategy (Table 1).

**Table 1:** Application of intercostal nerve cryoablation reported in the literature.

	Population pediatric vs. adult	Research type	INC—number	Control group	Control—number	INC intrathoracic vs. extrathoracic	Number of nerves cryoablated	Nerves	Temp of cryoprobe (°C)
Keller et al. (14)	Both	Retrospective review	26	TE	26	Intrathoracic	4 ICN Bilateral	T4–T7	−60
Harbaugh et al. (28)	Both	Retrospective review	19	TE	13	Intrathoracic	4–5 ICN Bilateral	NR	−60
Morikawa et al. (29)	Pediatric	Retrospective review	6	EEP	13	Intrathoracic	5 ICN Bilateral	NR	NR
Sujka et al. (30)	Pediatric	Retrospective review	9	TE or PCA	19	Intrathoracic	4 ICN Bilateral	T4–T7	NR
Parrado et al. (31)	Both	Retrospective review	45	MM EEP + MM	11 45	Intrathoracic	4 ICN Bilateral	T4–T7	−60
Graves et al. (13)	Both	Randomized control trial	10	TE	10	Intrathoracic	5 ICN Bilateral	NR	−60
Zobel et al. (32)	Both	Retrospective review	48	No control	NA	Intrathoracic	5 ICN Bilateral	NR	−60
Dekonenko et al. (33)	Both	Prospective review	35	TE PCA	32 33	Intrathoracic	4 ICN Bilateral	T4–T7	NR
Pilkington et al. (34)^a^	Pediatric	Retrospective	9	TE	20	Extrathoracic	NR	NR	−60 to −65
Rettig et al., (35)	Both	Retrospective review	40	TE	39	Extrathoracic	5 ICN Bilateral	T3–T7	−60
Torre et al., (57)	Both	Prospective review	7	No control	NA	Intrathoracic	6 ICN Bilateral	T3–T8	−70
Arshad et al. (36)	Pediatric	Retrospective Database	35	No-Cryo	140	Intrathoracic	NR	NR	NR
Aiken et al. (37)	Pediatric	Retrospective review	35	MM	38	Intrathoracic	5 ICN Bilateral	T3–T7	−60
Sun et al. (38)	Pediatric	Retrospective review	65	MM	119	Intrathoracic	5 ICN Bilateral	T3–T7	−65 to −70
Lai et al., (53)	Pediatric	Retrospective review	50	EEP MM	*n* = 50 *n* = 15	Intrathoracic	5 ICN Bilateral	T3–T7	<−40
Velayos et al. (39)	Pediatric	Retrospective review	NA	No control	NA	Preoperative percutaneous-guided cryoanalgesia conducted 48 h preoperative vs. day of surgery	NR	NR	NR
Difiore et al. (40)	Pediatric	Retrospective review	40	No control	NA	Intrathoracic	6 ICN Bilateral	T3–T8	−67
Song et al. (41)	Both	Retrospective review	38	TE	26	Intrathoracic	5–6 ICN Bilateral	NR	−70
Rettig et al., (54)^a^	Both	Retrospective review	19	TE	37	Extrathoracic	NR	NR	NR
Rettig et al., (58)	Both	Prospective	15	No control	NA	Intrathoracic	5 ICN Bilateral	T3–T7	NR
Rettig et al., (55)	Both	Retrospective review	15	INC + INB	15	Intrathoracic	5 ICN Bilateral	T3–T7	NR
Arshad et al. (42)	Pediatric	Retrospective review	20	No INC	15	Intrathoracic	NR	NR	NR
Clark et al. (43)	Pediatric	Retrospective review	75	MM	86	Intrathoracic	4 ICN Bilateral	T3–T6	−65
Fraser et al. (44)	Pediatric	Retrospective review	110	No control	NA	Intrathoracic	NR	NR	NR
Bundrant et al. (45)	Both	Retrospective review	35	MM	45	Intrathoracic	5 ICN Bilateral	T3–T7	−60
Lai et al., (59)	Pediatric	Retrospective review	350	INC Q1 vs. INC Q4	NA	Intrathoracic	4–6 ICN Bilateral	T4–T7 with T3 or T8 if possible	<−40
Cockrell et al. (46)	Both	Retrospective review	58	TE EEP	*n* = 78 *n* = 108	Intrathoracic	5 ICN Bilateral	NR	NR
Lai et al. (47)	Pediatric	Retrospective review	22	No control	NA	Intrathoracic	4 ICN Bilateral	T4–T7	−60
Downing et al. (48)	Pediatric	Prospective review	13	TE and NB	40	Intrathoracic	5 ICN Bilateral	T4–T8	NR
Akinboro et al. (49)	Both	Retrospective + prospective	17	PVB and R sided INC	12 9	Intrathoracic	5 ICN Bilateral	T3–T7	−69
Perez Holguin et al. (50)	Pediatric	Retrospective review	31	TE	127	Intrathoracic	5–6 ICN Bilateral	NR	−60
Gallardo et al. (56)	Both	Retrospective review	21	No control	NA	Intrathoracic	5 ICN Bilateral	T3–T7	−70
Zeineddin et al. (51)	Pediatric	Retrospective review	100	MM (PVB, ketamine)	98	Intrathoracic	5 ICN Bilateral	T3–T7	−60
Jaroszewski et al. (52)	Adult	Retrospective review	211	TE and EEP	90 428	Intrathoracic	6–7 ICN Bilateral	T3–T8 ± T9	−60

MM, multimodal pain regimen; EEP, elastomeric pain pump; PVB, paravertebral block; INB, intercostal nerve block.

^a^ Rettig et al. (54) performed INC during an open repair of PE.

INC was reported to be performed via an intrathoracic approach under thoracoscopic visualization in 90% of the cases. The number of intercostal nerves that were cryoablated ranged from eight to 12 between the intercostal space of T3–T8. Velayos et al. (39) reported performing INC preoperatively via a percutaneous approach. Almost all the researchers applied the cryoprobe for a single 2 min duration to each intercostal nerve, with one article reporting a single 1 min application of the cryoprobe. The temperature of the applied cryoprobe reached temperatures ranging from <−40 to −70°C. The operative times during the MIRPE with INC ranged from 60 to 153 min (Table 1).

### 3.1. Primary outcomes

The primary outcome discussed in the majority of the articles was hospital LOS and opioid usage (Table 2). The use of INC was associated with a significant decrease in LOS when compared with other analgesic strategies in 21 out of 22 articles (13, 14, 28–30, 32–38, 41–43, 46, 48–52, 54, 55). When comparing LOS between patients who had undergone INC vs. TE placement, Keller et al. (14) found that hospital LOS decreased from 5.8 days to 3.4 days. Other researchers have corroborated INCs effect on hospital LOS when compared with TE placement reporting a decreased LOS of 2–3.5 days (13, 28, 30, 33, 34, 41, 46, 48, 50, 52, 54). One study found no significant change in LOS when comparing INC with EPP (29); however, this study was possibly underpowered to find a statistical difference among cohorts, with only six patients receiving INC as part of their care. Alternatively, INC was found to reduce hospital LOS when compared with EPP in every other study that compared these two analgesic strategies (31, 38, 48, 53). EPP only provides analgesia while in place whereas INC provides a prolonged analgesic effect; in the prior studies, EPPs were typically in place for 48–72 h postoperatively and were discontinued prior to discharge. When INC is used in combination with a multimodal pain regimen, the researchers found that patients were able to be routinely discharged on post operative day (POD) 1 (33, 37, 40, 48, 49, 51, 56, 57). Recent publications have demonstrated the feasibility of a same-day discharge when INC is combined with a peripheral nerve block (PNB) with 65%–66% of patients being discharged on POD 0 (49, 55, 58).

**Table 2:** Hospital length of stay, opioid use, and pain scores of intercostal nerve cryoablation.

	Control group	Length of stay INC vs. control	In-hospital opioid usage INC vs. control	Discharge opioid INC vs. control	Pain scores INC vs. control	Hospital charges INC vs. control
Keller et al. (14)	TE	3.4 vs. 5.8 days*	Total hospital opioid 49 OME mg vs. 119 OME mg* Mean length of IV opioid 1.8 vs. 3.96 days*	NR	NR	NR
Harbaugh et al. (28)	TE	3 vs. 6 days*	Total hospital opioid 1.79 OME mg/kg vs 1.8 OME mg/kg	Discharge opioid 3.97 OME mg/kg vs. 5.81 OME mg/kg* Refills 11% vs. 38%	Median VAPS POD0 5 vs. 4 POD1 3 vs. 2	NR
Morikawa et al. (29)	EPP	2.2 vs. 3.7 days	Number of narcotic dosages 6.4 vs. 17.9 doses*	NR	Mean Hospital VAPS 2.2 vs. 3.7	NR
Sujka et al. (30)	TE or PCA	1.4 vs. 4.0 days*	Time to discontinuation of oral narcotics 8.2 vs. 18.2 days*	NR	Mean VAPS POD 0 4 vs. 6.5* POD1 5.4 vs. 5.1 POD2 3.3 vs. 6.1*	NR
Parrado et al. (31)	MM EEP + MM	NR	INC vs. MM vs. EEP + MM 237 OME mg vs. 466 OME mg vs. 347 OME mg*	NR	NR	NR
Graves et al. (13)	TE	3 vs. 5 days*	268 OME mg vs. 684 OME mg*	NR	Mean VAPS Day 1 3.1 vs. 3 Day 2 2.8 vs. 2.9 Week 2 2.2 vs. 2.1 1 month 2.5 vs. 1.9 3 months 1.3 vs. 1.1 1 year 1.3 vs. 1.1	NR
Dekonenko et al. (33)	TE PCA	INC vs. TE vs. PCA 1 vs. 4.3 vs. 4.2 days*	NR	NR	INC vs. TE vs. PCA Maximal VAPS POD0 6 vs. 7 vs. 8* POD1 5 vs. 5 vs. 5 POD2 6.5 vs. 6 vs. 5 POD 3 4.2 vs. 6 vs. 5 POD 4 4.5 vs. 5 vs. 5	NR
Pilkington et al. (34)	TE	4 vs. 6 days*	Intraoperative opioid 0.5 vs. 1.1 OME mg/kg* Total hospital Opioid 1.1 vs. 1.5 OME mg/kg	3.3 vs. 4.8 OME mg/kg	POD 2 3 vs. 4*	NR
Rettig et al. (35)	TE	2.5 vs. 5 days*	Total hospital Opioid 100 OME mg vs. 269 OME mg*	105 OME mg vs. 552 OME mg*	NR	Operating room $10,976 vs. $8,523* Total Hospitalization $15,976 vs. $18,335*
Arshad et al. (42)	No INC	2 vs. 3 days*	NR	NR	NR	NR
Aiken et al. (37)	MM	1 vs. 4 days*	Total opioid: 0−24 h 15 OME mg vs. 148 OME mg* 24−48 h 7.5 OME mg vs. 115 OME mg* Total admission 22.5 OME mg vs. 410 OME mg*	Discharge opioid 112.5 OME mg vs. 300 OME mg* Opioid refills 22.9% vs. 29.0%	Uncontrolled pain 0−24 h 0% vs. 29%* 24−48 h 8% vs. 7.9%	NR
Sun et al. (38)	MM	2 vs. 4 days*	Total hospital opioid 1.2 OME mg/kg vs. 5.0 OME mg/kg*	Discharge opioid 7.2 OME mg/kg vs. 11 OME mg/kg* Opioid use at 2-week follow-up 28% vs. 53%*	NR	NR
Lai et al., (59)	EPP MM	INC vs. EPP vs. MM 2 vs. 4 vs. 3 days*	Total hospital opioid 0.51 vs. 6.48 vs. 9.56 OME mg/kg* Per hospital day 0.28 vs. 1.9 vs. 2.77 OME mg/kg*	NR	Median hospital VAPS 4.68 vs. 4.48 vs. 5.49	NR
Song et al. (41)	TE	3 vs. 5 days*	Total hospital opioid 19 OME mg vs. 634 OME mg*	NR	Median hospital VAPS 2 vs. 5*	NR
Rettig et al., (54)	TE	2.8 vs. 6 days*	Total hospital opioid 91.6 OME mg vs. 779.9 OME mg*	Discharge opioid 147.1 mg OME vs. 511.7 mg OME*	NR	Operating room $18, 658 vs. $14,745* Total Hospitalization $33, 848 vs. $40,813*
Rettig et al., (55)	INC with INB	11.9 vs. 58.2 h*	NR	NR	NR	NR
Arshad et al. (42)	No INC	3 vs. 5 days*	Total hospital opioid 2.3 OME mg/kg vs. 4.9 OME mg/kg*	NR	NR	NR
Clark et al. (43)	MM	2 vs. 4 days*	Total PCA opioid 10.3 mg vs. 35.3 mg* Number of PRN IV opioid doses 0.4 vs. 1.3 doses* Oral opioid doses 4.2 vs. 8.6 doses*	NR	Mean hospital VAPS 2.2 vs. 2.4	NR
Cockrell et al. (46)	TE and EEP	2.4 vs. 4.1 days*	0−48 h postop 0.8 OME mg/kg vs. 1.9 OME mg/kg*	NR	PACU VAPS 6.0 vs. 7.7*	NR
Downing et al. (48)	TE	1 vs. 4 days*	POD1 1.47 vs. 1.96 OME/kg* Overall 3.12 vs. 6.35 OME/kg*	NR	Median hospital VAPS 6 vs. 7	NR
Akinboro et al. (49)	PVB with infusion PVB with infusion and R sided INC	INC vs. PVB w/o INC vs. PVB w INC 0.7 vs. 1.3 vs. 2.6* 65% of INC discharged on POD 0	INC vs. PVB w/o INC vs. PVB w INC POD0 0.92 vs. 9.47 vs. 0.62 OME mg/kg*	NR	INC vs. PVB w/o INC POD0 VAPS 2.3 vs. 4*	NR
Perez Holguin et al. (50)	TE	3.2 vs. 5.3 days*	Total hospital opioid 27.0 OME mg vs. 290 OME mg*	NR	NR	Total Hospitalization $24,742 vs. 21,621* Room and board $5,585 vs. $10,705* Operating room $6,198 vs. $3,916 Pharmacy $468 vs. $619 Radiology $317 vs. $259 Lab $81 vs. $26 Supplies and instruments $7,683 vs. $3,737* Other $1,952 vs. $1,619
Zeineddin et al. (51)	MM (PVB and ketamine)	1 day vs. 4 days*	Total hospital opioid 20.7 OME mg vs. 409 OME mg* 0.4 OME mg/kg vs. 7.5 OME mg/kg*	109 OME mg vs. 628 OME mg* 2 vs. 11.1 OME mg/kg	NR	Total hospitalization $14,072 vs. $21,021*
Jaroszewski et al. (52)	TE EEP	INC vs. TE vs. EEP 1.9 vs. 4.2 vs. 2.3 days*	INC vs. TE vs. EEP POD0 0.1 vs. 10.2 vs. 6.5 OME mg* POD1 10.8 vs. 37.6 vs. 55.4 OME mg* POD2 15.0 vs. 59.0 vs. 52.5 OME mg* POD3 7.5 vs. 60.0 vs. 45.0 OME mg*	NR	NR	NR

MM, multimodal pain regimen; EEP, elastomeric pain pump; PVB, paravertebral block; INB, intercostal nerve block; PACU, post anesthesia care unit.

* A statistically significant difference between groups, *p* < 0.05.

Opioid usage significantly decreased with INC use during MIRPE when compared with other analgesic strategies in all studies that reported opioid consumption (13, 14, 28, 30, 31, 35, 37, 38, 41–43, 46, 48–51, 53, 54). A majority of these studies reported opioid use in terms of OME milligrams and reported the total hospital OME milligram; however, most did not account for the LOS in the non-INC cohort when reporting opioid use (14, 28, 29, 31, 35, 38, 40–43, 48, 50, 51, 54). All researchers that compared opioid OME by individual hospital days reported a significant lower amount of opioid consumption among the INC cohort than those with other analgesic strategy (37, 46, 48, 49, 53). Of all articles comparing opioid consumption between an INC and non-INC cohort, all found equivocal or lower opioid consumption among those who had INC during MIRPE. The researchers found a significant decrease in the total OME prescription at discharge and duration of opioid use post-MIRPE when INC was utilized (28, 35, 37, 38, 43, 51, 54).

The effect of INC on visual analog pain scores (VAPS) varied between investigators, with less than half (5/11) of the articles finding a significant decrease in VAPS when INC was used (Table 2) (13, 28–30, 33, 41, 44, 46, 48, 49, 53). INC was associated with significantly lower VAPS only during the initial postoperative hospitalization. At the outpatient follow-up, there was no differences found in VAPS; however, VAPS were generally low following discharge in both the INC and non-INC cohorts.

### 3.2. Complications

The complications associated with INC were reported in 50% of the articles reviewed. The overall complication rate was either significantly lower or no difference was found between the INC and non-INC cohorts (28, 31, 35, 38, 40, 43, 45, 50, 51, 53, 54, 59). Postoperative urinary retention was found to improve with INC with rates ranging 4%–8% compared with the 14%–34% in those who did not have INC (38, 43).

Keller et al. 2016 and Sun et al. 2021 reported higher rates of clinically significant pectus bar migration requiring reoperation in patients with INC. In these studies, bar migration occurred in 8%–12% of patients who had INC; however, neither study provided a statistical comparison of bar migration between the INC group and a control. The bar migration was hypothesized to be secondary to an increased activity in patients with INC due to an improved pain control (14, 38). However, an increased bar migration has not been supported by other studies (28, 31, 59). In fact, the largest cohort study of INC in MIRPE, containing 350 patients, reported bar migration occurring in less than 1% of patients who received INC (59).

Neurosensory outcomes following the use of INC were reported in 16% of studies (13, 32, 37, 40, 51). A complete chest wall sensory return following cryoablation was reported to occur in 76.9%–100% of patients 1 year post-INC. No difference in neuropathic pain was found between patients with INC and those with an alternative analgesic strategy (13, 51). Zobel et al. conducted a retrospective review comparing neuropathic pain between adolescent and adult patients using a validated neuropathic pain survey. They found that neuropathic pain was more common in adults (>21 years of age) (32). In children, the incidence rate of neuropathic pain was 0% at 12 months (40). While these studies demonstrate a relatively low risk for developing persistent sensory loss or chronic neuropathic pain, most studies were retrospective in nature, creating an inherent bias in their findings. No articles discussed in detail how chest wall sensory examinations were performed or validated. In addition, only one article compared sensory outcomes between INC and a control group. Graves et al. conducted a randomized control trial between INC and the use of TE during MIRPE. In this study, they reported the sensory outcomes between each cohort at different intervals postoperatively. All patients with INC (*n* = 10) had reported chest wall sensory loss at their 2-week postoperative exam; interestingly, 20% (2/10) of the patients without INC also had some degree of chest wall sensory loss noted 2 weeks postoperatively. A complete chest wall sensory return was noted in both the INC and non-INC cohort prior to the study completion (13). This finding suggests that some sensory loss may be attributable to surgical technique; however, this study was underpowered to truly compare sensory loss and recovery between INC and MIRPE.

### 3.3. Economic impact

The majority of articles that discuss total hospital costs and charges found that INC is associated with a decrease in cost when compared with other analgesic strategies (35, 37, 50, 51, 54). The median overall cost of MIRPE with INC ranged from $14,072 to $33,848 compared with $18,335–$40,813 MIRPE without INC. All investigators who included an itemized cost analysis found that the use of INC was associated with a greater operative room cost (35, 50, 54). One of five studies found that the use of INC was associated with higher hospital cost. Perez Holguin et al. conducted a retrospective review comparing hospital cost between TE use from 2002 to 2020 and INC use from 2017 to 2020. They found an overall increased hospital cost from $21,621 to $24,742 when INC was used compared with TE; the largest contributor to cost with INC was the intraoperative charge of $6,198 vs. $3,916 for TE. However, in their cost analysis, they failed to account for inflation and operative technique, i.e., number of pectus bars implanted, bar stabilization between groups (50). Similarly, Aiken et al. performed a cost analysis of INC compared with a standardized pain control cohort between 2016 and 2019. The total hospital costs were adjusted to 2018 dollars to standardize monetary value across each study year. They found that the total hospital cost was lower in the INC cohort, $21,924, compared with the non-INC cohort, $23,694 (Table 2) (37).

## 4. Discussion

**Figure 1:**
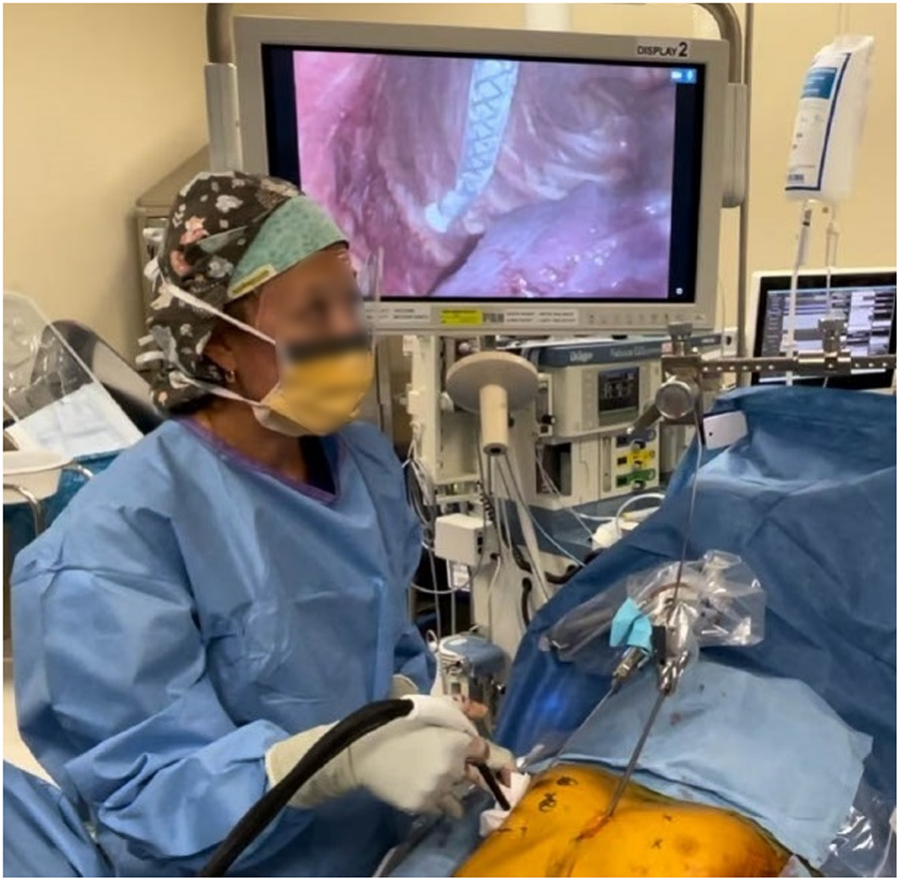
Intraoperative use of intercostal nerve cryoablation. The cryoprobe is inserted into the chest under direct thoracoscopic visualization. The cryoprobe is placed just inferior to the costal rig and applied for a 2 min duration at a temperature of −60°C. The probe is placed at least 4 cm from the spinal column to avoid injury to the sympathetic chain. An “ice ball” is formed at the tip of the cryoprobe during the freezing process. A lung isolation strategy is used to avoid pulmonary tissue thermal injury using a dual lumen endotracheal tube. A surgical laparotomy pad is used to protect the skin from inadvertent thermal injury as pictured.

Since the introduction of INC during MIRPE, it has consistently shown to decrease hospital LOS and opioid usage among children and adolescents. In addition, INC has a favorable side effect profile with minimal associated morbidity. INC is routinely performed on the bilateral chest wall under direct visualization, using a single lung ventilatory strategy, between the intercostal nerves T3 and T8 at a temperature of −40°C to −60°C for a 2-min duration (Figure 1). The cryoprobe is allowed to actively rewarm to a temperature of −4°C prior to removal from the chest wall to avoid tissue fracture/injury. Care is taken to avoid inadvertent contact of the cryoprobe and lung tissue to avoid thermal pulmonary injury and delayed pneumothorax; in addition, the anesthesiologist continues contralateral single lung ventilation for 3 min from the last INC to avoid thermal injury from the chest wall.

The largest series of patients undergoing the MIRPE with INC was reported in 2022 (53). This study was a retrospective review that captured 350 patients who had undergone INC between December 2017 and August 2021. The mean age of the study cohort was 15.7 years with a Haller index of 5.4 and correction index of 35.2. The patients were divided into time-based quartiles determined by their operative dates; the patient outcomes were compared between the first and forth quartile. The authors found a decreased hospital LOS, total OME milligram, and OME milligram per day between the first and forth time-based quartiles. In addition, the patients had a relatively low morbidity with <1% having pectus bar migration and <5% requiring a 90-day readmission and a 90-day wound infection. Despite these findings, this study lacked a control arm that had not undergone INC as part of their pain strategy.

Those who do not support the use of INC during MIRPE in children and adolescents argue that while INC has been shown to decrease hospital LOS and opioid consumption, it lacks data supporting long-term safety and efficacy. They further cite that no studies have adequately compared INC with the erector spinae block, which is associated with a short hospital LOS and low opioid use and may spare adolescent patients from the possible neurosensory and neuropathic pain complications of INC (23). Future prospective studies are warranted to compare the long-term neurosensory effects of INC and to determine the incidence rate of chronic neuropathic pain.

In addition to neurosensory outcomes, there is a paucity of literature regarding the effect of INC on the psychosocial and physiologic quality of life of the patient. The repair of PE has been shown to have a significant improvement in both self-perception and physiologic status of the patient (60–62). In the current literature, only one article addressed pulmonary functions following MIRPE with INC. Lai et al. (53) demonstrated that INC did not worsen the pulmonary function of the patient, as measured by incentive spirometry, when compared with the use of an elastomeric pain pump. Research is needed to address the effects that INC has on the psychosocial and cardiopulmonary performance outcomes following MIRPE. Furthermore, an investigation of the impact that INC has on postoperative patient activity is warranted as some have reported unacceptably high rates of pectus bar migration following INC use (14, 38).

In the current literature that reviews the use of INC during the repair of PE, most studies were performed retrospectively leading to inherent bias and limitations (63). Of the studies that were conducted in a prospective manner, all were possibly underpowered without discussion of a power calculation and the largest number of patients receiving INC in any study being 35 (13, 33, 48, 49, 57, 58). Among the prospective studies, INC was only compared against TE, PCA, and intercostal nerve blocks. In addition, there has only been one randomized control trial comparing INC with any other analgesic strategy. Again, this study was limited by a small sample of five patients, who had undergone INC as part of the MIRPE (13). The paucity of appropriately powered prospective studies ultimately limits the conclusions that can be drawn with regard to the true effect that the INC has on patient outcomes. Future prospective randomized control trials are needed to compare INC with other analgesic strategies.

## 5. Conclusion

INC is an effective analgesic strategy following the MIPRE, with its use known to decrease hospital LOS and opioid consumption with minimal morbidity.

## Data Availability

The original contributions presented in the study are included in the article/Supplementary Material, further inquiries can be directed to the corresponding author.

## References

[1] Biavati M Kozlitina J Alder AC Foglia R McColl RW Peshock RM Prevalence of pectus excavatum in an adult population-based cohort estimated from radiographic indices of chest wall shape. PLoS One. (2020) 15(5):e0232575. 10.1371/journal.pone.0232575 32379835 PMC7205298

[2] Obermeyer RJ Goretsky MJ. Chest wall deformities in pediatric surgery. Surg Clin North Am. (2012) 92(3):669–84. 10.1016/j.suc.2012.03.001 22595715

[3] Frawley G Frawley J Crameri J. A review of anesthetic techniques and outcomes following minimally invasive repair of pectus excavatum (Nuss procedure). Paediatr Anaesth. (2016) 26(11):1082–90. 10.1111/pan.12988 27510834

[4] Fokin AA Steuerwald NM Ahrens WA Allen KE. Anatomical, histologic, and genetic characteristics of congenital chest wall deformities. Semin Thorac Cardiovasc Surg. (2009) 21(1):44–57. 10.1053/j.semtcvs.2009.03.001 19632563

[5] Brochhausen C Turial S Müller FKP Schmitt VH Coerdt W Wihlm JM Pectus excavatum: history, hypotheses and treatment options. Interact Cardiovasc Thorac Surg. (2012) 14(6):801–6. 10.1093/icvts/ivs045 22394989 PMC3352718

[6] Nuss D Obermeyer RJ Kelly RE. Pectus excavatum from a pediatric surgeon's perspective. Ann Cardiothorac Surg. (2016) 5(5):493–500. 10.21037/acs.2016.06.04 27747183 PMC5056929

[7] Mao YZ Tang ST Li S. Comparison of the Nuss versus Ravitch procedure for pectus excavatum repair: an updated meta-analysis. J Pediatr Surg. (2017) 52(10):1545–52. 10.1016/j.jpedsurg.2017.05.028 28606386

[8] Kelly RE Shamberger RC Mellins RB Mitchell KK Lawson ML Oldham K Prospective multicenter study of surgical correction of pectus excavatum: design, perioperative complications, pain, and baseline pulmonary function facilitated by internet-based data collection. J Am Coll Surg. (2007) 205(2):205–16. 10.1016/j.jamcollsurg.2007.03.027 17660066

[9] Stroud AM Tulanont DD Coates TE Goodney PP Croitoru DP. Epidural analgesia versus intravenous patient-controlled analgesia following minimally invasive pectus excavatum repair: a systematic review and meta-analysis. J Pediatr Surg. (2014) 49(5):798–806. 10.1016/j.jpedsurg.2014.02.072 24851774 PMC5315444

[10] St. Peter SD Weesner KA Weissend EE Sharp SW Valusek PA Sharp RJ Epidural vs. patient-controlled analgesia for postoperative pain after pectus excavatum repair: a prospective, randomized trial. J Pediatr Surg. (2012) 47(1):148–53. 10.1016/j.jpedsurg.2011.10.040 22244408

[11] Burton DMH Boretsky KR. A comparison of paravertebral nerve block catheters and thoracic epidural catheters for postoperative analgesia following the Nuss procedure for pectus excavatum repair. Paediatr Anaesth. (2014) 24(5):516–20. 10.1111/pan.12369 24612096

[12] Choudhry DK Brenn BR Sacks K Reichard K Lonnqvist PA. Continuous chest wall ropivacaine infusion for analgesia in children undergoing Nuss procedure: a comparison with thoracic epidural. Paediatr Anaesth. (2016) 26(6):582–9. 10.1111/pan.12904 27061848

[13] Graves CE Moyer J Zobel MJ Mora R Smith D O’Day M Intraoperative intercostal nerve cryoablation during the Nuss procedure reduces length of stay and opioid requirement: a randomized clinical trial. J Pediatr Surg. (2019) 54(11):2250–6. 10.1016/j.jpedsurg.2019.02.057 30935731 PMC6920013

[14] Keller BA Kabagambe SK Becker JC Chen YJ Goodman LF Clark-Wronski JM Intercostal nerve cryoablation versus thoracic epidural catheters for postoperative analgesia following pectus excavatum repair: preliminary outcomes in twenty-six cryoablation patients. J Pediatr Surg. (2016) 51(12):2033–8. 10.1016/j.jpedsurg.2016.09.034 27745867

[15] Brain HS. Three types of nerve injury. Brain. (1943) 66(4):237–88. 10.1093/brain/66.4.237

[16] Gordon T. Peripheral nerve regeneration and muscle reinnervation. Int J Mol Sci. (2020) 21(22):1–24. 10.3390/ijms21228652 PMC7697710 33212795

[17] Chen P Xianhua P Bonaldo P. Role of macrophages in Wallerian degeneration and axonal regeneration after peripheral nerve injury. Acta Neuropathol. (2015) 3:605–18. 10.1007/s00401-015-1482-4 26419777

[18] Shafiq M Sethi J Ali MS Ghori UK Saghaie T Folch E. Pleural cryobiopsy: a systematic review and meta-analysis. Chest. (2020) 157(1):223–30. 10.1016/j.chest.2019.09.023 31610161

[19] Talsma J Kusakavitch M Lee D Niederhauser C Palmer B Ozgediz D Forgotten branch of the intercostal nerve: implication for cryoablation nerve block for pectus excavatum repair. J Pediatr Surg. (2023) S0022-3468(23):00294–4. 10.1016/j.jpedsurg.2023.05.006 37286412

[20] Chen SY Mack SJ Stein JE Kelley-Quon LI Kim ES. Intercostal nerve cryoablation is associated with reduced opioid use in pediatric oncology patients. J Surg Res. (2023) 283:377–84. 10.1016/j.jss.2022.11.004 36427448 PMC10756229

[21] Gologorsky R Ewbank C Idowu O Kim S. Use of cryoanalgesia as a postoperative pain management for open pectus carinatum repair. Pediatr Surg Int. (2021) 37(1):179–81. 10.1007/s00383-020-04768-z 33112997

[22] Das B Sadhasivam S. Response to intercostal nerve cryoablation versus thoracic epidural catheters for postoperative analgesia following pectus excavatum repair. J Pediatr Surg. (2017) 52(6):1076. 10.1016/j.jpedsurg.2017.01.069 28302362

[23] Chidambaran V Garcia VF Brown RL. Are we ready for cryoablation in children undergoing Nuss procedures? Anesth Analg. (2022) 134(4):881–4. 10.1213/ANE.0000000000005857 35299214

[24] McCoy N Hollinger L. Cryoanalgesia and lung isolation: a new challenge for the Nuss procedure made easier with the EZ-Blocker™. Front Pediatr. (2021) 9:791607. 10.3389/fped.2021.791607 34912765 PMC8667069

[25] Daemen JHT de Loos ER Vissers YLJ Bakens MJAM Maessen JG Hulsewé KWE. Intercostal nerve cryoablation versus thoracic epidural for postoperative analgesia following pectus excavatum repair: a systematic review and meta-analysis. Interact Cardiovasc Thorac Surg. (2020) 31(4):486–98. 10.1093/icvts/ivaa151 32929487

[26] Singhal NR Jerman JD. A review of anesthetic considerations and postoperative pain control after the Nuss procedure. Semin Pediatr Surg. (2018) 27(3):156–60. 10.1053/j.sempedsurg.2018.05.010 30078486

[27] Pechetov AA Lednev AN Makov MA Chlan TN. Intercostal nerve cryoablation in correction of pectus excavatum in adults. Khirurgiia (Sofiia). (2021) 5:14–9. 10.17116/hirurgia202105114 33977693

[28] Harbaugh CM Johnson KN Kein CE Jarboe MD Hirschl RB Geiger JD Comparing outcomes with thoracic epidural and intercostal nerve cryoablation after Nuss procedure. J Surg Res. (2018) 231:217–23. 10.1016/j.jss.2018.05.048 30278932

[29] Morikawa N Laferriere N Koo S Johnson S Woo R Puapong D. Cryoanalgesia in patients undergoing Nuss repair of pectus excavatum: technique modification and early results. J Laparoendosc Adv Surg Tech A. (2018) 28(9):1148–51. 10.1089/lap.2017.0665 29672193

[30] Sujka J Benedict LA Fraser JD Aguayo P Millspaugh DL St Peter SD. Outcomes using cryoablation for postoperative pain control in children following minimally invasive pectus excavatum repair. J Laparoendosc Adv Surg Tech A. (2018) 28(11):1383–6. 10.1089/lap.2018.0111 29927703

[31] Parrado R Lee J McMahon LE Clay C Powell J Kang P The use of cryoanalgesia in minimally invasive repair of pectus excavatum: lessons learned. J Laparoendosc Adv Surg Tech A. (2019) 29(10):1244–51. 10.1089/lap.2019.0203 31259649

[32] Zobel MJ Ewbank C Mora R Idowu O Kim S Padilla BE. The incidence of neuropathic pain after intercostal cryoablation during the Nuss procedure. Pediatr Surg Int. (2020) 36(3):317–24. 10.1007/s00383-019-04602-1 31760443

[33] Dekonenko C Dorman RM Duran Y Juang D Aguayo P Fraser JD Postoperative pain control modalities for pectus excavatum repair: a prospective observational study of cryoablation compared to results of a randomized trial of epidural vs. patient-controlled analgesia. J Pediatr Surg. (2020) 55(8):1444–7. 10.1016/j.jpedsurg.2019.09.021 31699436

[34] Pilkington M Harbaugh CM Hirschl RB Geiger JD Gadepalli SK. Use of cryoanalgesia for pain management for the modified Ravitch procedure in children. J Pediatr Surg. (2020) 55(7):1381–4. 10.1016/j.jpedsurg.2019.09.016 31672412

[35] Rettig RL Rudikoff AG Lo HYA Shaul DB Banzali FM Conte AH Cryoablation is associated with shorter length of stay and reduced opioid use in pectus excavatum repair. Pediatr Surg Int. (2021) 37(1):67–75. 10.1007/s00383-020-04778-x 33210165

[36] Arshad SA Hatton GE Ferguson DM Li LT Austin MT Tsao KJ. Cryoanalgesia enhances recovery from minimally invasive repair of pectus excavatum resulting in reduced length of stay: a case-matched analysis of NSQIP-pediatric patients. J Pediatr Surg. (2021) 56(7):1099–102. 10.1016/j.jpedsurg.2021.03.017 33853733

[37] Aiken TJ Stahl CC Lemaster D Casias TW Walker BJ Nichol PF Intercostal nerve cryoablation is associated with lower hospital cost during minimally invasive Nuss procedure for pectus excavatum. J Pediatr Surg. (2021) 56(10):1841–5. 10.1016/j.jpedsurg.2020.10.009 33199059 PMC8053720

[38] Sun RC Mehl SC Anbarasu CR Portuondo JI Espinoza AF Whitlock R Intercostal cryoablation during Nuss procedure: a large volume single surgeon’s experience and outcomes. J Pediatr Surg. (2021) 56(12):2229–34. 10.1016/j.jpedsurg.2021.03.006 33853732

[39] Velayos M Alonso M Delgado-Miguel C Estefanía-Fernández K Muñoz-Serrano AJ Santamaría MVL Percutaneous cryoanalgesia: a new strategy for pain management in pectus excavatum surgery. Eur J Pediatr Surg. (2022) 32(1):73–9. 10.1055/s-0041-1740555 34942673

[40] DiFiore JW Robertson JO Chhabada S DeRoss AL Hossain MS Rincon-Cruz L Next day discharge after the Nuss procedure using intercostal nerve cryoablation, intercostal nerve blocks, and a perioperative ERAS pain protocol. J Pediatr Surg. (2022) 57(2):213–8. 10.1016/j.jpedsurg.2021.10.034 34823843

[41] Song SH Moon DH Shim YH Jung H Lee S. Limited cryoablation reduces hospital stay and opioid consumption compared to thoracic epidural analgesia after minimally invasive repair of pectus excavatum. Medicine (Baltimore). (2022) 101(31):E29773. 10.1097/MD.0000000000029773 35945758 PMC9351910

[42] Arshad SA Ferguson DM Garcia EI Hebballi NB Buchanan AC Tsao KJ. Cryoanalgesia is associated with decreased postoperative opioid use in minimally invasive repair of pectus excavatum. J Surg Res. (2022) 271:1–6. 10.1016/j.jss.2021.10.011 34814047

[43] Clark RA Jacobson JC Singhal A Alder AC Chung DH Pandya SR. Impact of cryoablation on pectus excavatum repair in pediatric patients. J Am Coll Surg. (2022) 234(4):484–92. 10.1097/XCS.0000000000000103 35290267

[44] Fraser JA Briggs KB Svetanoff WJ Aguayo P Juang D Fraser JD Short and long term outcomes of using cryoablation for postoperative pain control in patients after pectus excavatum repair. J Pediatr Surg. (2022) 57(6):1050–5. 10.1016/j.jpedsurg.2022.01.051 35277249

[45] Bundrant NT Sayrs LW Ostlie D Lee J Egan C Molitor M Infectious complications of intercostal nerve cryoablation mediated by perioperative hypothermia during pediatric Nuss procedure. J Pediatr Surg. (2022) 57(6):1083–6. 10.1016/j.jpedsurg.2022.01.044 35232599

[46] Cockrell HC Hrachovec J Schnuck J Nchinda N Meehan J. Implementation of a cryoablation-based pain management protocol for pectus excavatum. J Pediatr Surg. (2023) 58(7):1239–45. 10.1016/j.jpedsurg.2023.01.059. Available at: https://pubmed.ncbi.nlm.nih.gov/36894442/ (Accessed May 14, 2023). 36894442

[47] Lai K Eldredge RS Nguyen M Padilla BE McMahon LE. Initial outcomes using cryoablation in surgical management of slipping rib syndrome. J Pediatr Surg. (2023) 58(8):1430–4. 10.1016/j.jpedsurg.2022.12.031. Available at: https://pubmed.ncbi.nlm.nih.gov/36737261/ (Accessed May 14, 2023). 36737261

[48] Downing L Ramjist JK Tyrrell A Tsang M Isaac L Fecteau A. Development of a five point enhanced recovery protocol for pectus excavatum surgery. J Pediatr Surg. (2023) 58(5):822–7. 10.1016/j.jpedsurg.2023.01.028 36788057

[49] Akinboro S John R Reyna T Davis R Ayoub C Sangster R A pilot study of multi-modal pain management for same-day discharge after minimally invasive repair of pectus excavatum (Nuss procedure) in children. Pediatr Surg Int. (2023) 39(1):159. 10.1007/s00383-023-05429-7 36967421 PMC10040230

[50] Perez Holguin RA DeAngelo N Sinha A Shen C Tsai AY. Cost and outcomes of intercostal nerve cryoablation versus thoracic epidural following the Nuss procedure. J Pediatr Surg. (2023) 58(4):608–12. 10.1016/j.jpedsurg.2022.12.011 36646539

[51] Zeineddin S Goldstein SD Linton S DeBoer C Alayleh A Ortiz I Effectiveness of one minute per level intercostal nerve cryoablation for postoperative analgesia after surgical correction of pectus excavatum. J Pediatr Surg. (2023) 58(1):34–40. 10.1016/j.jpedsurg.2022.09.032 36283847

[52] Jaroszewski DE Bostoros P Farina JM Botros MM Aly MR Peterson M Evolution of pain control for adult pectus excavatum repair. Ann Thorac Surg. (2023) S0003-4975(23):00570–2. 10.1016/j.athoracsur.2023.04.044 37279827

[53] Lai K Lee J Notrica DM Egan JC McMahon LE Molitor MS Intercostal nerve cryoablation in minimally invasive repair of pectus excavatum: effect on pulmonary function. J Laparoendosc Adv Surg Tech A. (2022) 32(12):1244–8. 10.1089/lap.2022.0242 36350702

[54] Rettig RL Yang CJ Ashfaq A Sydorak RM. Cryoablation is associated with shorter length-of-stay and reduced opioid use after the Ravitch procedure. J Pediatr Surg. (2022) 57(7):1258–63. 10.1016/j.jpedsurg.2022.02.040 35379492

[55] Rettig RL Rudikoff AG Annie Lo HY Lee CW Vazquez WD Rodriguez K Same-day discharge following the Nuss repair: a comparison. J Pediatr Surg. (2022) 57(1):135–40. 10.1016/j.jpedsurg.2021.09.023 34670678

[56] Cadaval Gallardo C Martínez J Bellía-Munzon G Nazar M Sanjurjo D Toselli L Thoracoscopic cryoanalgesia: a new strategy for postoperative pain control in minimally invasive pectus excavatum repair. Cir Pediatr. (2020) 33(1):11–5. Available at: https://pubmed.ncbi.nlm.nih.gov/32166917/ (Accessed May 14, 2023). 32166917

[57] Torre M Mameli L Bonfiglio R Guerriero V Derosas L Palomba L A new device for thoracoscopic cryoanalgesia in pectus excavatum repair: preliminary single center experience. Front Pediatr. (2020) 8:614097. 10.3389/fped.2020.614097 33585365 PMC7874221

[58] Rettig RL Rudikoff AG Lo HYA Lee CW Vazquez WD Rodriguez K Same day discharge for pectus excavatum—is it possible? J Pediatr Surg. (2022) 57(9):34–8. 10.1016/j.jpedsurg.2021.02.007 33678403

[59] Lai K Notrica DM McMahon LE Kang P Molitor MS Egan JC Cryoablation in 350 Nuss procedures: evolution of hospital length of stay and opioid use. J Pediatr Surg. (2022) 58(8):1435–9. 10.1016/j.jpedsurg.2022.10.051. Available at: https://pubmed.ncbi.nlm.nih.gov/36494205/ (Accessed May 14, 2023). 36494205

[60] Kuru P Bostanci K Ermerak NO Bahadir AT Afacan C Yuksel M. Quality of life improves after minimally invasive repair of pectus excavatum. Asian Cardiovasc Thorac Ann. (2015) 23(3):302–7. 10.1177/0218492314553442 25293414

[61] Kuru P Cakiroglu A Er A Ozbakir H Cinel AE Cangut B Pectus excavatum and pectus carinatum: associated conditions. Family history, and postoperative patient satisfaction. Korean J Thorac Cardiovasc Surg. (2016) 49(1):29–34. 10.5090/kjtcs.2016.49.1.29 26889443 PMC4757394

[62] Lawson ML Cash TF Akers R Vasser E Burke B Tabangin M A pilot study of the impact of surgical repair on disease-specific quality of life among patients with pectus excavatum. J Pediatr Surg. (2003) 38(6):916–8. 10.1016/S0022-3468(03)00123-4 12778393

[63] Jager KJ Tripepi G Chesnaye NC Dekker FW Zoccali C Stel VS. Where to look for the most frequent biases? Nephrology. (2020) 25(6):435–41. 10.1111/nep.13706 32133725 PMC7318122

