# Supplementary material for: Intercostal nerve cryoablation therapy for the repair of pectus excavatum: a systematic review
Source: Front Surg. 2023 Aug 24;10:1235120. doi: 10.3389/fsurg.2023.1235120 (PMC10484532; doi:10.3389/fsurg.2023.1235120)
Supplement: Supplementary file 1 [file Table1.docx]

Table 1.

|  | Population  Pediatric vs adult | Research type | INC – number | control group | Control –number | INC  intrathoracic vs extrathoracic | Number of nerves cryoablated | Nerves | Temp of cryoprobe |
| --- | --- | --- | --- | --- | --- | --- | --- | --- | --- |
| Keller 2016 | Both | Retrospective review | 26 | TE | 26 | intrathoracic | 4 ICN Bilateral | T4 - T7 | -60°C |
| Harbaugh 2018 | Both | Retrospective review | 19 | TE | 13 | intrathoracic | 4 - 5 ICN Bilateral | NR | -60°C |
| Morikawa 2018 | Pediatric | Retrospective  review | 6 | EEP | 13 | intrathoracic | 5 ICN Bilateral | NR | NR |
| Sujka 2018 | Pediatric | Retrospective review | 9 | TE or PCA | 19 | intrathoracic | 4 ICN Bilateral | T4 - T7 | NR |
| Parrado 2019 | Both | Retrospective review | 45 | MM EEP + MM | 11 45 | intrathoracic | 4 ICN Bilateral | T4 - T7 | -60°C |
| Graves 2019 | Both | Randomized Control Trial | 10 | TE | 10 | intrathoracic | 5 ICN Bilateral | NR | -60°C |
| Zobel 2020 | Both | Retrospective review | 48 | No control | NA | intrathoracic | 5 ICN Bilateral | NR | -60°C |
| Dekoneko 2020 | Both | Prospective review | 35 | TE PCA | 32 33 | intrathoracic | 4 ICN Bilateral | T4 - T7 | NR |
| *Pilkington 2020 | Pediatric | Retrospective | 9 | TE | 20 | extrathoracic | NR | NR | -60 to -65°C |
| Rettig 2021 | both | Retrospective review | 40 | TE | 39 | extrathoracic | 5 ICN Bilateral | T3 - T7 | -60°C |
| Torre 2021 | both | Prospective review | 7 | No control | NA | intrathoracic | 6 ICN Bilateral | T3 - T8 | - 70°C |
| Arshad 2021 | Pediatric | Retrospective Database | 35 | No-Cryo | 140 | intrathoracic | NR | NR | NR |
| Aiken 2021 | Pediatric | Retrospective review | 35 | MM | 38 | intrathoracic | 5 ICN Bilateral | T3 - T7 | -60°C |
| Sun 2021 | Pediatric | Retrospective review | 65 | MM | 119 | intrathoracic | 5 ICN Bilateral | T3 - T7 | -65 to -70°C |
| Lai 2022 | Pediatric | Retrospective review | 50 | EEP  MM | n=50 n=15 | intrathoracic | 5 ICN Bilateral | T3 - T7 | <-40°C |
| Velayos 2022 | Pediatric | Retrospective review | NA | No control | NA | Preoperative percutaneous guided cryoanlagesia conducted 48 hours preoperative vs day of surgery | NR | NR | NR |
| Difiore 2022 | Pediatric | Retrospective review | 40 | No control | NA | intrathoracic | 6 ICN Bilateral | T3 - T8 | -67°C |
| Song 2022 | Both | Retrospective review | 38 | TE | 26 | intrathoracic | 5 - 6 ICN Bilateral | NR | -70°C |
| *Rettig 2022 | Both | Retrospective review | 19 | TE | 37 | extrathoracic | NR | NR | NR |
| Rettig 2022 | Both | Prospective | 15 | No control | NA | Intrathoracic | 5 ICN Bilateral | T3 - T7 | NR |
| Rettig 2022 | Both | Retrospective review | 15 | INC + INB | 15 | Intrathoracic | 5 ICN Bilateral | T3 - T7 | NR |
| Arshad 2022 | Pediatric | Retrospective review | 20 | No INC | 15 | intrathoracic | NR | NR | NR |
| Clark 2022 | pediatric | Retrospective review | 75 | MM | 86 | intrathoracic | 4 ICN Bilateral | T3 - T6 | -65°C |
| Fraser 2022 | Pediatric | Retrospective review | 110 | No control | NA | intrathoracic | NR | NR | NR |
| Bundrant 2022 | Both | Retrospective review | 35 | MM | 45 | intrathoracic | 5 ICN Bilateral | T3 - T7 | - 60°C |
| Lai 2022 | Pediatric | Retrospective review | 350 | INC Q1 vs INC Q4 | NA | intrathoracic | 4 - 6 ICN Bilateral | T4 - T7 with T3 or T8 if possible | <-40°C |
| Cockrell 2023 | both | Retrospective review | 58 | TE EEP | n=78 n=108 | intrathoracic | 5 ICN Bilateral | NR | NR |
| Lai 2023 | Pediatric | Retrospective review | 22 | No control | NA | intrathoracic | 4 ICN Bilateral | T4 - T7 | -60°C |
| Downing 2023 | Pediatric | Prospective review | 13 | TE and NB | 40 | intrathoracic | 5 ICN Bilateral | T4 - T8 | NR |
| Akinboro 2023 | Both | Retrospective + prospective | 17 | PVB & R sided INC | 12 9 | intrathoracic | 5 ICN Bilateral | T3 - T7 | -69°C |
| Holguin 2023 | Pediatric | Retrospective review | 31 | TE | 127 | intrathoracic | 5 - 6 ICN Bilateral | NR | -60°C |
| Gallardo 2023 | Both | Retrospective review | 21 | No control | NA | intrathoracic | 5 ICN Bilateral | T3 - T7 | -70°C |
| Zeineddin 2023 | Pediatric | Retrospective review | 100 | MM (PVB, ketamine) | 98 | intrathoracic | 5 ICN Bilateral | T3 - T7 | -60°C |
| Jaroszewski 2023 | Adult | Retrospective review | 211 | TE & EEP | 90  428 | Intrathoracic | 6 – 7 ICN Bilateral | T3 - T8 ± T9 | -60°C |

Abbreviations: TE – thoracic epidural, PCA – patient controlled anesthesia, MM – multimodal pain regimen, EEP – elastomeric pain pump, PVB – paravertebral block, INB – intercostal nerve block, INC – intercostal nerve cryoablation. * Rettig et al. 2022 performed INC during open repair of PE.
