# Supplementary material for: Intercostal nerve cryoablation therapy for the repair of pectus excavatum: a systematic review
Source: Front Surg. 2023 Aug 24;10:1235120. doi: 10.3389/fsurg.2023.1235120 (PMC10484532; doi:10.3389/fsurg.2023.1235120)
Supplement: Supplementary file 2 [file Table2.docx]

Table 2. Comparison of hospital length of stay, opioid consumption and pain scores between intercostal nerve cryoablation (INC) and control.

|  | Control group | Length of stay  INC vs Control | In hospital opioid usage  INC vs Control | Discharge Opioid  INC vs Control | Pain scores  INC vs Control | Hospital charges  INC vs Control |
| --- | --- | --- | --- | --- | --- | --- |
| Keller 2016 | TE | 3.4 vs 5.8 days* | **Total hospital opioid** 49 OME mg vs 119 OME mg*  **Mean length of IV opioid** 1.8 vs 3.96 days * | NR | NR | NR |
| Harbaugh 2018 | TE | 3 vs 6 days* | **Total hospital opioid**  1.79 OME mg/kg vs  1.8 OME mg/kg | **Discharge opioid** 3.97 OME mg/kg vs 5.81 OME mg/kg* **Refills** 11% vs 38% | **Median VAPS** **POD0** 5 vs 4 **POD1**  3 vs 2 | NR |
| Morikawa 2018 | EPP | 2.2 vs 3.7 days | **Number of narcotic dosages** 6.4 vs 17.9 doses* | NR | **Mean Hospital VAPS** 2.2 vs 3.7 | NR |
| Sujka 2018 | TE or PCA | 1.4 vs 4.0 days* | **Time to discontinuation of oral narcotics** 8.2 vs 18.2 days* | NR | **Mean VAPS** **POD 0**  4 vs 6.5* **POD1**  5.4 vs 5.1  **POD2**  3.3 vs 6.1 * | NR |
| Parrado 2019 | MM EEP + MM | NR | **INC vs MM vs EEP + MM**  237 OME mg vs 466 OME mg vs 347 OME mg* | NR | NR | NR |
| Graves 2019 | TE | 3 vs 5 days* | 268 OME mg vs 684 OME mg* | NR | **Mean VAPS** **Day 1**  3.1 vs 3  **Day 2**  2.8 vs 2.9  **Week 2**  2.2 vs 2.1  **1 month**  2.5 vs 1.9  **3 month**  1.3 vs 1.1  **1 year**  1.3 vs 1.1 | NR |
| Dekoneko 2020 | TE PCA | **INC vs TE vs PCA**  1 vs 4.3 vs 4.2 days* | NR | NR | **INC vs TE vs PCA**  **Maximal VAPS** **POD0**  6 vs 7 vs 8 * **POD1**  5 vs 5 vs 5  **POD2**  6.5 vs 6 vs 5  **POD 3** 4.2 vs 6 vs 5  **POD 4**  4.5 vs 5 vs 5 | NR |
| Pilkington 2020 | TE | 4 vs 6 days* | **Intraoperative opioid**  0.5 vs 1.1 OME mg/kg*  **Total hospital Opioid**  1.1 vs 1.5 OME mg/kg | 3.3 vs 4.8 ome mg/kg | **POD 2**  3 vs 4* | NR |
| Rettig 2021 | TE | 2.5 vs 5 days * | **Total hospital Opioid** 100 OME mg vs 269 OME mg* | 105 OME mg vs 552 OME mg* | NR | **Operating room**  $10,976 vs $8,523*  **Total Hospitalization**  $15,976 vs $18,335* |
| Arshad 2021 | No-INC | 2 vs 3 days* | NR | NR | NR | NR |
| Aiken 2021 | MM | 1 vs 4 days* | Total opioid: **0-24 hour**  15 OME mg vs 148 OME mg*  **24-48 hours**  7.5 OME mg vs 115 OME mg*  **Total admission**  22.5 OME mg vs 410 OME mg* | **Discharge opioid** 112.5 OME mg vs 300 OME mg*  **Opioid refills**  22.9% vs 29.0% | **Uncontrolled pain**  **0-24 hours** 0% vs 29%*  **24-48 hours**  8% vs 7.9% | NR |
| Sun 2021 | MM | 2 vs 4 days* | **Total hospital opioid** 1.2 OME mg/kg vs 5.0 OME mg/kg * | **Discharge opioid** 7.2 OME mg/kg vs 11 OME mg/kg* **Opioid use at 2 week** follow up 28% vs 53%* | NR | NR |
| Lai 2022 | EPP MM | INC vs EPP vs MM  2 vs 4 vs 3 days* | **Total hospital opioid**  0.51 vs 6.48 vs 9.56 OME mg/kg *  **Per hospital day** 0.28 vs 1.9 vs 2.77 OME mg/kg * | NR | **Median hospital VAPS** 4.68 vs 4.48 vs 5.49 | NR |
| Song 2022 | TE | 3 vs 5 days* | **Total hospital opioid** 19 OME mg vs 634 OME mg* | NR | **Median hospital VAPS** 2 vs 5 * | NR |
| Rettig 2022 | TE | 2.8 vs 6 days* | **Total hospital opioid**  91.6 OME mg vs 779.9 OME mg* | **Discharge opioid**  147.1 mg OME vs 511.7 mg OME* | NR | **Operating room**  $18, 658 vs $14,745*  **Total Hospitalization**  $33, 848 vs $40,813* |
| Rettig 2022 | INC with INB | 11.9 vs 58.2 hours* | NR | NR | NR | NR |
| Arshad 2022 | No INC | 3 vs 5 days* | **Total hospital opioid** 2.3 OME mg/kg vs 4.9 OME mg/kg* | NR | NR | NR |
| Clark 2022 | MM | 2 vs 4 days * | **Total PCA opioid** 10.3 mg vs 35.3 mg* **Number of PRN IV opioid doses** 0.4 vs 1.3 doses* **Oral opioid doses**  4.2 vs 8.6 doses* | NR | **Mean hospital VAPS** 2.2 vs 2.4 | NR |
| Cockrell 2023 | TE & EEP | 2.4 vs 4.1 days* | **0-48 hr postop** 0.8 OME mg/kg vs 1.9 OME mg/kg* | NR | **PACU VAPS** 6.0 vs 7.7 * | NR |
| Downing 2023 | TE | 1 vs 4 days* | **POD1** 1.47 vs 1.96 OME/kg* **Overall** 3.12 vs 6.35 OME/kg* | NR | **Median hospital VAPS**  6 vs 7 | NR |
| Akinboro 2023 | PVB with infusion  PVB with infusion & R sided INC | **INC vs PVB w/o INC vs PVB w INC**  0.7 vs 1.3 vs 2.6* 65% of INC discharged on POD 0 | **INC vs PVB w/o INC vs PVB w** INC **POD0** 0.92 vs 9.47 vs 0.62 OME mg/kg* | NR | **INC vs PVB w/o INC** **POD0 VAPS** 2.3 vs 4 * | NR |
| Holguin 2023 | TE | 3.2 vs 5.3 days* | **Total hospital opioid** 27.0 OME mg vs 290 OME mg* | NR | NR | **Total Hospitalization**  $24,742 vs 21,621 *  **Room and board**  $5,585 vs $10,705 *  **Operating room**  $6,198 vs $3,916  **Pharmacy**  $468 vs $619  **Radiology**  $317 vs $259  **Lab**  $81 vs $26  **Supplies & instruments**  $7683 vs $3737*  **Other**  $1,952 vs $1,619 |
| Zeineddin 2023 | MM  (PVB and ketamine) | 1 day vs 4 days* | **Total hospital opioid** 20.7 OME mg vs 409 OME mg* 0.4 OME mg/kg vs 7.5 OME mg/kg* | 109 OME mg vs 628 OME mg* 2 vs 11.1 OME mg/kg | NR | **Total Hospitalization**  $14,072 vs $21,021* |
| Jaroszewski  2023 | TE  EEP | **INC vs TE vs EEP**  1.9 vs 4.2 vs 2.3 days* | **INC vs TE vs EEP**  **POD0**   - 1. vs 10.2 vs 6.5 OME mg*   **POD1**  10.8 vs 37.6 vs 55.4 OME mg*  **POD2**  15.0 vs 59.0 vs 52.5 OME mg*  **POD3**  7.5 vs 60.0 vs 45.0 OME mg* | NR | NR | NR |

Abbreviations: TE – thoracic epidural, PCA – patient-controlled anesthesia, MM – multimodal pain regimen, EEP – elastomeric pain pump, PVB – paravertebral block, INB – intercostal nerve block, INC – intercostal nerve cryoablation. * Signifies a significant statiscially significant difference between groups, p<0.05.
